# Inhibition of NF-κB activation by 5-lipoxygenase inhibitors protects brain against injury in a rat model of focal cerebral ischemia

**DOI:** 10.1186/1742-2094-3-12

**Published:** 2006-05-11

**Authors:** Manu Jatana, Shailendra Giri, Mubeen A Ansari, Chinnasamy Elango, Avtar K Singh, Inderjit Singh, Mushfiquddin Khan

**Affiliations:** 1 Department of Pediatrics, Medical University of South Carolina, Charleston, SC 29425, USA; 2 Department of Pathology and Laboratory medicine, Ralph H. Johnson VA Medical Center Charleston, SC 29425, USA

## Abstract

**Background:**

Stroke is one of the leading causes of death worldwide and a major cause of morbidity and mortality in the United States of America. Brain ischemia-reperfusion (IR) triggers a complex series of biochemical events including inflammation. Leukotrienes derived from 5-lipoxygenase (5-LOX) cause inflammation and are thus involved in the pathobiology of stroke injury.

**Methods:**

To test the neuroprotective efficacy of 5-LOX inhibition in a rat model of focal cerebral IR, ischemic animals were either pre- or post-treated with a potent selective 5-LOX inhibitor, (N- [3-[3-(-fluorophenoxy) phenyl]-1-methyl-2-propenyl]-*N*-hydroxyurea (BW-B 70C). They were evaluated at 24 h after reperfusion for brain infarction, neurological deficit score, and the expression of 5-LOX. Furthermore, the mechanism and the anti-inflammatory potential of BW-B 70C in the regulation of nuclear factor kappa B (NF-κB) and inflammatory inducible nitric oxide synthase (iNOS) were investigated both *in vivo *and *in vitro*.

**Results and discussion:**

Both pre- and post-treatment with BW-B 70C reduced infarctions and improved neurological deficit scores. Immunohistochemical study of brain sections showed IR-mediated increased expression of 5-LOX in the neurons and microglia. BW-B 70C down-regulated 5-LOX and inhibited iNOS expression by preventing NF-κB activation. Two other structurally different 5-LOX inhibitors were also administered post IR: caffeic acid and 2, 3, 5-trimethyl-6- [12-hydroxy-5, 10-dodecadiynyl]-1, 4-benzoquinone (AA-861). As with BW-B 70C, they provided remarkable neuroprotection. Furthermore, in vitro, BW-B 70C inhibited lipopolysaccharide (LPS) mediated nitric oxide production, iNOS induction and NF-κB activation in the BV2 microglial cell line. Treating rat primary microglia with BW-B70C confirmed blockage of LPS-mediated translocation of the p65 subunit of NF-κB from cytosol to nucleus.

**Conclusion:**

The study demonstrates the neuroprotective potential of 5-LOX inhibition through down-regulation of NF-κB in a rat model of experimental stroke.

## Introduction

Cerebral ischemia-reperfusion (IR) triggers lipid peroxidation and inflammation, which exacerbate injury. Recognition of inflammatory components involved in stroke has expanded the list of potential targets for therapy [1]. They include inducible nitric oxide synthase (iNOS), cyclooxygenase-2 (COX-2), nuclear factor kappa B (NF-κB) and 5-lipoxygeanse (5-LOX) [2,3]. 5-LOX is the key enzyme in leukotriene biosynthesis [4]. It translocates to the nuclear membrane upon stimulation, where it co-localizes with 5-LOX activating protein (FLAP) and cytosolic phospholipase A_2 _(cPLA_2_) [5]. This event converts arachidonic acid to leukotrienes. Emerging data implicate both 5-LOX and FLAP in the disease process of cerebral ischemia [6]. Increased leukotriene levels and 5-LOX expression have been documented in stroke patients [7]. Also, FLAP has recently been identified as the first common gene associated with higher risk in atherosclerosis and stroke [8].

5-LOX-mediated reactive oxygen species (ROS) generation has been implicated in the activation of NF-κB [9,10]. Recently, we have demonstrated that PLA_2 _and 5-LOX are involved in lipopolysaccharide (LPS)-induced iNOS gene expression via dependent and independent NF-κB pathways in glial cells [11]. NF-κB is an important transcription factor that plays a pivotal role in mediating inflammatory response to pro-inflammatory cytokines and ROS in animal models of experimental stroke [10,12]. In unstimulated cells, p50:p65 is sequestered in the cytoplasm by inhibitory proteins known as NF-κB inhibitors (IκBs). Upon stimulation, IκB is phosphorylated by an upstream IκB kinase (IKK), which leads to its ubiquitination and proteosomal degradation. This process liberates p50:p65, which translocates to the nucleus and induces transcription of several genes, including iNOS. In ischemia, the p65 subunit is recognized to play an important role in regulation of inflammation [13]. It has also been shown that P65 interaction with 5-LOX activates NF-κB [14].

In the present study, we used a 5-LOX inhibitor, N- [3-[3-(-fluorophenoxy) phenyl]-1-methyl-2-propenyl]-*N*-hydroxyurea (BW-B 70C), in a rat model of focal cerebral IR. BW-B 70C demonstrated a neuroprotective role through inhibition of both 5-LOX and NF-κB. It is a potent and a selective inhibitor of 5-LOX *in vitro *and *in vivo *with a long half-life and high oral bioavailability. Other potent 5-LOX inhibitors, caffeic acid and 2, 3, 5-trimethyl-6- [12-hydroxy-5, 10-dodecadiynyl]-1, 4-benzoquinone (AA-861) confirmed the neuroprotective efficacy of 5-LOX inhibition. A similar protective effect of AA-861 has been reported in gerbils after transient ischemia [15].

Our observations document that 5-LOX inhibition protects against IR injury in rats via down-regulation of the inflammatory mediators NF-κB and iNOS. Thus, inhibiting the 5-LOX/NF-κB pathway holds therapeutic potential to attenuate inflammation-mediated brain injury after an ischemic stroke.

## Methods

### Reagents and cell culture

Dulbecco's Modified Eagle's Medium (DMEM) with glucose, L-glutamine and sodium pyruvate was purchased from Mediatech Inc. (Herndon, VA), Fetal Bovine Serum (FBS) and Hank's balanced salt solution were obtained from Life Technologies (Carlsbad, CA). Lipopolysaccharide (LPS; 0111:B4)) from *Escherichia coli*, and MTT (3-(4, 5-dimethylthiazol-2-yl)-2, 5-diphenyltetrazolium bromide) were obtained from Sigma-Aldrich Chemical Corporation (St. Louis, MO). Antibody against 5-LOX was purchased from Cayman Chemical (Ann Arbor, Michigan). Antibody against p65, p50, iNOS, NSE and β-actin were purchased from Santa Cruz Biotechnology, Inc. (Santa Cruz, CA), and RCA-1, (ricinus communis agglutinin-1) was purchased from Vector Laboratories, (Burlingame, CA). Anti-cow GFAP was purchased from DakoCytomation California Inc. (Carpinteria, CA). BW-B 70C was purchased from Tocris (Ellisville, MO). Caffeic acid and AA-861 were purchased from Biomol (Plymouth Meeting, PA). The enhanced chemiluminescence (ECL) detecting reagent was from Amersham Pharmacia Biotech (Arlington Heights, IL), and the luciferase assay system was from Promega (Madison, WI,). NF-κB-luciferase chemiluminescence kit was purchased from Clontech (Palo Alto, CA). IQ Sybr Green Supermix was purchased from Bio-Rad (Hercules, CA).

### Animals

Male Sprague-Dawley rats weighing 240–260 g (Harlan Laboratories, Wilmington, MA) were used in this study. All animal procedures were approved by the Medical University of South Carolina (MUSC) Animal Review Committee and animals received humane care in compliance with MUSC's experimental guidelines and the National Research Council's criteria for humane care (Guide for the Care and Use of Laboratory Animals).

### Experimental groups

The animals were divided into three groups: i) Control (sham), ii) Ischemia/reperfusion (Vehicle) and (iii) Treated (treated with 5-LOX inhibitors). In the treatment group, the rats were given 5-LOX inhibitor, dissolved in sterile DMSO (15 μl) intravenously (IV) in the jugular vein either before ischemia or at reperfusion. Three structurally different 5-LOX inhibitors used were: BW-B 70C, 2.0 mg/kg 30 minutes before ischemia or 3.0 mg/kg at reperfusion; AA-861, 3.0 mg/kg at reperfusion; caffeic acid, 3.0 mg/kg of body weight at reperfusion. The rats in the vehicle and sham groups were administered the same volume of DMSO alone.

### Transient focal cerebral ischemia model

Rats were subjected to middle cerebral artery occlusion (MCAO) as described previously [16,17] with slight modification. Briefly, rats were anesthetized with intraperitoneal injection of xylazine (10 mg/kg body weight) and ketamine hydrochloride (100 mg/kg). With the aid of a dissecting microscope, the right common, internal and external carotid arteries were exposed and the vagus nerve separated carefully. Next the external carotid artery (ECA) was isolated and ligated. A 4-0-monofilament nylon suture (Harvard Apparatus, MA) was inserted through the ECA into the internal carotid artery until a mild resistance was felt, to occlude the middle cerebral artery (MCA) [18]. Animals were kept under constant conditions for 20 minutes of ischemia. At the end of the ischemic period, the monofilament was withdrawn and the common carotid artery clamp was removed. The animals were then allowed to recover from anesthesia. During surgery, the whole body temperature was maintained at 37.0 ± 0.5°C using a heating pad, and monitored using a rectal temperature probe.

### Measurement of regional cerebral blood flow (CBF)

The occlusion of MCA and reperfusion were monitored by measuring the regional CBF using laser Doppler flow meter (Oxyflo, Oxford Optronics, England and Periflux system 5000; Perimed Inc., Sweden). For measurement of the blood flow, a needle-shaped laser probe was placed over the skull (on right side) at 1 mm posterior and 4.0 mm lateral to the bregma. Baseline of CBF was obtained before MCAO. CBF was monitored continuously during ischemia (20 min) with a criterion of < 20% of baseline blood flow remaining after MCAO. Reperfusion was confirmed by laser Doppler readings.

### Measurement of physiologic parameters

The physiological variables were measured before and after 30 min of reperfusion and are presented in Table 1. The rectal temperature was monitored and maintained at about 37 to 37.8°C. Body temperature was monitored by a rectal probe and maintained at about 37 ± 0.5 °C by a homeothermic blanket control unit (Harvard Apparatus, Holliston, MA). Cranial temperature was measured by HSE Plugsys TAM-D (Harvard Apparatus). Blood gases and blood pH were measured by pH/blood gas analyzer iSTAT (Heska, Fort Collins, CO). Mean blood pressure (MBP) was measured using a XBP1000 NIBP system (Kent Scientific, Torrington, CT). It is non-invasive computerized tail-cuff system and uses automated inflation/deflation pump. Blood glucose levels were measured in plasma using Quantichrom glucose assay kit (Bioassay systems, Haywood, CA).

**Table 1:** Physiologic parameters

	Vehicle		Caffeic acid		AA-861		BW-B 70C	
	Basal	30 min Rep	Basal	30 min Rep	Basal	30 min Rep	Basal	30 min Rep

Rectal Temp (°C)	37.2 ± 0.3	36.8 ± 0.2	37.6 +0.2	36.5 ± 0.3	37.4 ± 0.4	37.3 ± 0.2	37.3 ± 0.3	37.4 ± 0.2
Cranial Temp (°C)	37.0 ± 0.5	37.3 ± 0.7	36.8 ± 0.2	36.9 ± 0.3	37.4 ± 0.3	36.9 ± 0.4	36.9 ± 0.2	37.1 ± 0.4
PCO2 (mm Hg)	56.9 ± 7.6	55.7 ± 8.0	51.4 ± 9.2	52.4 ± 6.9	55.9 ± 9.0	53.8 ± 9.7	58.5 ± 6.4	52.6 ± 7.8
PO2 (mm Hg)	41.5 ± 7.8	44.0 ± 5.2	48.5 ± 8.2	39.5 ± 8.9	43.5 ± 8.4	37.0 ± 5.6	45.5 ± 6.3	38.5 ± 6.5
MBP (mm Hg)	106 ± 14	101 ± 10	102 ± 23	112 ± 10	109 ± 13	107 ± 12	117 ± 19	121 ± 14
Glucose (mg/dL)	190.5 ± 15.2	214.8 ± 18.6	210.5 ± 12.5	191.6 ± 12.9	201.8 ± 11.9	181.6 ± 16.8	190.5 ± 17.5	202.6 ± 10.9
PH	7.3 ± 0.1	7.3 ± 0.2	7.3 ± 0.1	7.3 ± 0.1	7.2 ± 0.2	7.3 ± 0.1	7.3 ± 0.1	7.3 ± 0.2

Measurements were performed before MCAO (base) and at 30 min of reperfusion after drug administration as described in Methods. All measurements other than temperature were performed in blood. Data are presented as mean ± SD for n = 3 in each group. Measurements were also performed for sham group. No significant differences were observed among the groups. Basal, 30 min before MCAO; MBP, mean blood pressure; Rep, reperfusion; Temp, temperature

### Neurological evaluation

Neurological deficit in the animals was determined by an observer blinded to the identity of the groups, and was assessed at 24 h of reperfusion. The scoring was based on method of Huang et al. [19] as follows: 0, no observable neurological deficit (normal); 1, failure to extend left forepaw on lifting the whole body by tail (mild); 2, circling to the contralateral side but normal posture at rest (moderate); 3, leaning to the contralateral side at rest (severe); 4, no spontaneous motor activity (very severe).

### Measurement of ischemic infarct and image acquisition

Infarct volume was evaluated as previously described [17]. Briefly, after 24 h of reperfusion, the brains were quickly removed and placed in ice-cold saline for 5 min and then coronal sections were obtained at 2-mm intervals from the frontal pole. The slices were incubated in 2% 2, 3, 5-triphenyltetrazolium chloride (TTC) (Sigma, MO) dissolved in saline for 15 min at 37°C. The brain sections were fixed by immersion in 10% formalin. The image of infarct area was acquired in Photoshop 7.0 (Adobe Systems) and quantified using Scion image image-analysis software (Scion Corporation). The volume of infarction was obtained from the product of average slice thickness (2 mm) and sum of infarction area in all brain slices. Infarct values were corrected for edema as described by Swanson et al [20]. Edema in this model contributed less than 10% of total infarction.

### Immunohistochemistry

Protein expression was detected by immunohistochemical analysis. Paraffin embedded sections of brain tissues were stained for 5-LOX, RCA-1 (Ricinus communis agglutinin-1), GFAP (Glial fibrillary acidic protein), iNOS, p65 and NSE (Neuron-specific enolase). BV2 cells and rat primary microglia were stained for p65 protein expression. In brief, the brain tissue sections were deparafinised and rehydrated in sequential gradations of alcohol. After antigen unmasking in unmasking solution (Vector Labs, CA), sections were cooled and washed three times for two minutes each in PBS. Sections were immersed for 10 min in 3% hydrogen peroxide to eliminate endogenous peroxidase activity and blocked in 1% bovine serum albumin for 1 hour. Sections were incubated overnight with respective primary antibody (1:100 dilution in blocking buffer). After washing in PBS containing 0.1% Tween-20, sections were incubated with the appropriate fluorophore tagged secondary antibody (1:100 dilution in blocking buffer) (Vector Labs, CA). Fluorescence was visualized under the microscope. All the sections were analyzed using a Zeiss Olympus Microscope and images were captured using a Kontron Digital Camera. At least ten different fields were recorded for each measurement and a representative image was presented in figures. Images were captured and processed in Adobe Photoshop 7.0 (Adobe Systems, CA) and were adjusted by using the brightness and contrast level and unmasking tools to enhance image clarity.

### Maintenance of cell lines and preparation of rat primary microglia

BV2 cells were maintained in DMEM (4.5 g glucose/L) supplemented with 10% fetal FBS plus antibiotics and induced with stimuli as indicated. The cell line was kindly provided by Dr Michael McKinney of Mayo Clinic (Jacksonville, FL, USA). Primary microglia were prepared from rat cerebral tissue as described previously [21]. Briefly, after 10 days of culture, astrocytes were separated from microglia and oligodendrocytes by shaking for 24 h in an orbital shaker at 240 rpm. The microglia were plated onto poly-lysine-coated plates for one hour, subsequently the unattached cells were removed. For induction of nitric oxide (NO), cells were stimulated with LPS under serum-free conditions.

### Assay for NO synthesis

NO production was determined in cell culture supernatants by measurement of nitrite, a stable reaction product formed from released NO and molecular oxygen. Briefly, 100 μl of culture supernatant was made to react with 100 μl of Griess reagent and incubated at room temperature for 15 minutes for optimal reaction product formation. The absorbance of the assay samples was measured spectrophotometrically at 570 nm using SpectraMax 190 (Molecular Devices, CA). NO concentrations were calculated from a standard curve derived from the reaction of NaNO_2 _(sodium nitrite) used as a standard in the assay.

### Preparation of nuclear extracts

Nuclear extracts were prepared from treated and untreated cells as described previously [22] based on modified method of Dignam and coworkers [23]. At stipulated time points after treatment, cells were harvested, washed twice with ice-cold PBS. Cells were then lysed in 400 μl of buffer A (containing: 10 mM HEPES, pH 7.9, 10 mM KCl, 2 mM MgCl_2_, 0.5 mM dithiothreitol, 1 mM PMSF, 5 μg/ml aprotinin, 5 μg/ml pepstatin A, and 5 μg/ml leupeptin containing 0.1% Nonidet P-40) for 15 minutes on ice. Cells were vortexed vigorously for 15 seconds, and then centrifuged at 20,000 × *g *for 30 seconds. The pelleted nuclear fraction was then resuspended in 40 μl of buffer B (20 mM HEPES, pH 7.9, 25% (v/v) glycerol, 0.42 M NaCl, 1.5 mM MgCl_2_, 0.2 mM EDTA, 0.5 dithiothreitol, 1 mM PMSF, 5 μg/ml aprotinin, 5 μg/ml pepstatin A, and 5 μg/ml leupeptin) and kept on ice for 30 minutes. Lysates were centrifuged at 20,000 × *g *for 10 minutes. Supernatants containing the nuclear proteins were diluted with 20 μl of modified buffer C (20 mM HEPES, pH 7.9, 20% (v/v) glycerol, 0.05 M KCl, 0.2 mM EDTA, 0.5 mM dithiothreitol, and 0.5 mM PMSF) and were processed for immunoblotting immediately or stored at -70°C until further use.

### Immunoblot analysis

For immunoblotting, after incubation in the presence and absence of stimuli, the cells were scraped off, washed with Hank's buffer, and sonicated in 50 mM Tris-HCl (pH 7.4) containing protease inhibitors (1 mM PMSF, 5 μg/ml aprotinin, 5 μg/ml antipain, 5 μg/ml pepstatin A, and 5 μg/ml leupeptin). Proteins were resolved by SDS-PAGE and transferred onto nitrocellulose membranes. The membranes were blocked for 1 h in 5% nonfat dry milk TTBS (20 mM Tris, 500 mM NaCl, and 0.1% Tween 20, pH 7.5) and incubated overnight at 4°C in primary antibody containing 5% nonfat dry milk. The blots were then washed four times with TTBS (5 min/wash) and incubated for 45 minutes at room temperature with HRP-conjugated secondary antibody at a dilution of 1:5000. The blots were then washed three times in TTBS and once in 0.1 M PBS (pH 7.4) at room temperature; the desired protein was detected with ECL, per the manufacturer's specifications (Amersham Pharmacia Biotech).

### Transfection studies

Transfection of cells was done as described previously [22]. Plasmids were purified using the endotoxin-free plasmid midi-prep kit (Qiagen, Santa Clarita, CA, USA). For transient transfections, BV2 cells were seeded in 6-well plates and grown to 60–80% confluence in DMEM medium with 5% FBS (without antibiotics), and were transfected using FuGene reagent (Promega) with 1.5 μg of NF-κB-luc reporter construct vector or insertless expression vector (pBluescript). 24 h after transfection, cells were maintained in serum-free medium overnight and then treated with LPS and/or BW-B 70C. Finally, 24 h later, luciferase activity was measured in cell lysates prepared using lysis buffer (Promega) as per the manufacture's protocol.

### cDNA synthesis and real time PCR analysis

cDNA Synthesis and real time PCR analysis was carried out with certain modifications of method described earlier [24]. Total RNA from brain tissue was isolated using Trizol reagent (Gibco BRL, Carlsbad, CA) as per manufacturer's instructions. Single-stranded cDNA was synthesized from pooled RNA samples of rat brains from three similarly treated rats by using the superscript preamplification system (Life Technologies, Carlsbad, CA). Quantitative real-time PCR was performed with the Bio-Rad (Hercules, CA) iCycler iQ Real-Time PCR Detection System as per conditions described previously [24]. Briefly, primer sets were designed and synthesized from Integrated DNA Technologies (IDT, Coralville, IA). The primer sequences were: GAPDH, forward primer, 5'-cctacccccaatgtatccgttgtg-3', reverse primer, 5'-ggaggaatgggagttgctgttgaa-3'; iNOS, forward primer, 5'-ggaagaggaacaactactgctggt-3', reverse primer, 5'-gaactgagggtacatgctggagc-3'. Thermal cycling conditions were as follows: activation of iTaq DNA polymerase at 95°C for 10 minutes, followed by 40 cycles of amplification at 95 °C for 30 seconds and 55–57.5°C for 1 minute. The normalized expression of target gene with respect to GAPDH was computed for all samples by using Microsoft Excel data spreadsheets.

### Statistical analysis

Statistical analysis was performed using software Graphpad Prism 3.0, unless stated otherwise. Values are expressed as mean ± SD of n determinations or as mentioned. Comparisons among means of groups were made with a two-tailed Student's *t-test *for unpaired variables. Multiple comparisons were performed using one-way ANOVA followed by Bonferroni test. p values less than 0.05 were considered significant.

## Results

### Treatment with 5-LOX inhibitors improves brain infarction and neurological score after IR injury

**Figure 1:**
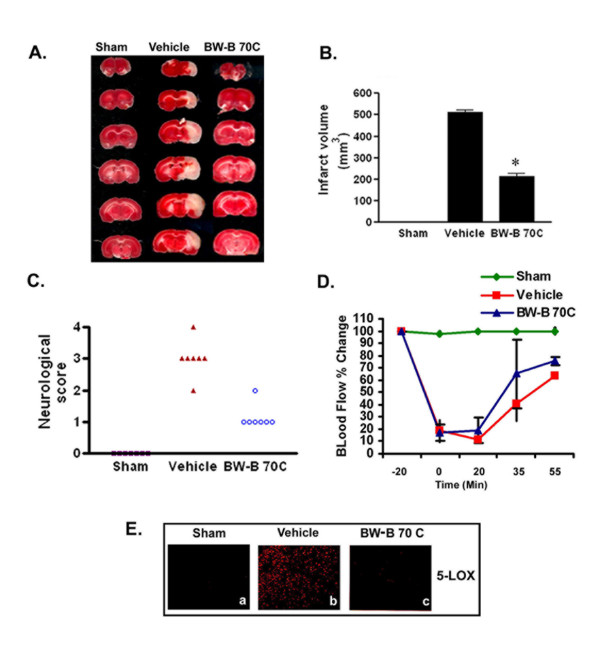
**Pretreatment with BW-B 70C protects the brain from infarction and improves neurological score. **(A) Photograph showing effect of BW-B 70C on TTC-stained sections, (B) Effect of BW-B 70C on infarct volume (measured in six serial coronal sections arranged from cranial to caudal regions), (C) Effect of BW-B 70C on neurological score and (D) Effect of BW-B 70C on regional cerebral blood flow (CBF). Changes in CBF were not significantly different after ischemia between the untreated (vehicle) and treatment (BW-B 70C) groups. (E) Photomicrograph of expression of 5-LOX (n = 4) at 24 h reperfusion after 20 min MCAO (magnification X200). Data for infarct volume (n = 7) and blood flow (n = 4) are presented as means ± SD. *p < 0.001 vs. vehicle. Data for neurological deficit score (n = 7) are presented as individual data points.

Pretreatment with 5-LOX inhibitor BW-B 70C (2 mg/kg) reduced infarct volume and improved neurological deficit score (Fig. 1A–C) recorded at 24 h reperfusion after 20 min MCAO in rats. The animals were monitored for changes in regional CBF before during and after occlusion (Fig. 1D). Changes in the CBF were not significantly different after ischemia between the untreated (vehicle) and BW-B 70C treated groups. CBF measurements indicated that all rats were subjected to a similar degree of ischemia (>80% drop in CBF compared to baseline). Fig. 1A shows representative TTC stained sections from sham; vehicle and BW-B 70C treated animals, showing that the treatment reduced infarction. As seen in Fig. 1B, BW-B 70C treated animals had reduced infarct volume 215.0 ± 35.0 mm^3 ^compared with vehicle group (512.2 ± 30.5 mm^3^). Furthermore, significant neuroprotection was observed even when BW-B 70C (3 mg/kg) was administered at the time of reperfusion after ischemia (infarct volume: 205.2 ± 8.9 mm^3^) as shown in Table 2A. Other structurally different 5-LOX inhibitors, caffeic acid and AA-861, provided similar degrees of protection when administered at the time of reperfusion. They reduced the infarct volumes to 229.5 ± 18.5 and 210.4 ± 20.5, respectively (Table 2A). Neurological deficit scores were evaluated at 24 h after reperfusion (Fig. 1C and Table 2B), and were consistent with the changes observed in infarct volume. Pre-treatment with BW-B 70C improved the neurological score to median1.0 compared to the vehicle group median 3.0, (Fig. 1C). Even after ischemia, treatment with 5-LOX inhibitors reduced neurological score to median1.0 compared to the vehicle group (median 3.0) as shown in Table 2B. The use of an effective dose of 5-LOX inhibitors is based on maximal brain protection (infarct volume) at lowest dose determined from a study carried out separately for each inhibitor. Administration of either 2 mg/kg or 3 mg/kg of BW-B 70C prior to ischemia had similar effect on reduction of infarctions. However, the treatment with 2 mg/kg BW-B 70C after ischemia was less effective compared to 3 mg/kg. The selected dose had no significant effects on physiologic parameters (blood gases, cranial temperature, mean blood pressure, blood glucose and pH) as shown in Table 1.

**Table 2:** Infarct volume, infarct area and neurological score in untreated (vehicle) and 5-LOX inhibitor-treated rats at 24 h reperfusion after 20 min MCAO.

**A.Infarct volume and infarct area**				
	**Group**	**N**	**Infarct volume (mm**^3^**)**	**Infarct area (%)**
	**Vehicle**	7	522.5 ± 11.2	41.3 ± 10.5
	**Caffeic acid**	7	229.5 ± 18.5*	12.8 ± 1.9*
	**AA-861**	7	210.4 ± 20.5*	11.3 ± 2.9*
	**BW-B 70C**	7	205.2 ± 8.9*	11.8 ± 2.1*

**B.Neurological Score**	**Number of animals = 7**			
	
	**Vehicle**	**Caffeic acid**	**AA-861**	**BW-B 70C**

	**Frequencies**			

**0**	0	0	0	0
**1**	0	4	4	5
**2**	0	3	2	2
**3**	6	0	1	0
**4**	1	0	0	0

**Median**	**3**	**1**	**1**	**1**

Infarct volume and infarct area (A) and neurological deficit score (B) were measured at 24 h of reperfusion after 20 min MCAO. Animals were treated either with 5-LOX inhibitors dissolved in DMSO alone (vehicle) at reperfusion as described in Methods. Data in Table B represent number of animals showing corresponding neurological score (ranging from 0 to 4 as detailed in Methods). Data are expressed as mean ± SD for infarct area and infarct volume and as range and median for neurological deficit score. *p < 0.001 vs. vehicle.

Postmortem studies have shown that brain sections from stroke patients are positive for 5-LOX expression [7]. To investigate the increased expression of the 5-LOX enzyme after IR injury, and whether administration of BW-B 70C reduces the 5-LOX expression, we subjected rat brain tissue sections to immunohistochemistry (Fig. 1E, a–c). There was increased expression of 5-LOX in the ipsilateral hemisphere of brain tissue sections at 24 h after reperfusion (Fig. 1E, b), which was reduced by the administration of BW-B 70C (Fig. 1E, c). The mechanisms of 5-LOX inhibition by a non-redox type inhibitor BW-B 70C are not clear.

**Figure 2:**
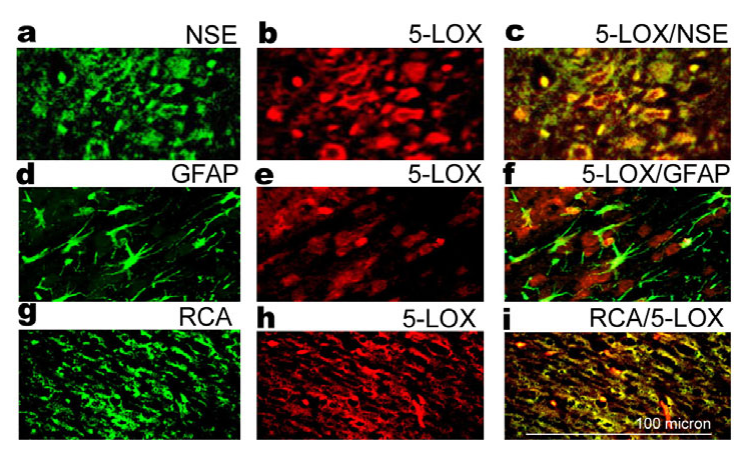
**5-LOX is expressed in neurons and microglia/macrophages. **Co-localization of expression of (a) NSE, (d) GFAP; (g) RCA; and 5-LOX (b,e,h) at 24 h reperfusion after 20 min MCAO. Yellow fluorescence indicates co-localization of 5-LOX/NSE (c) and 5-LOX/RCA (i). 5-LOX/GFAP (f) showed very few yellow-fluorescent structures. Figures are representative of similar results obtained from three different sections of three different animals in each group (Magnification 400 ×).

Co-localization of 5-LOX expression in neurons and microglia/macrophage in brain after IR injury.As inferred from the data above (Fig. 1E, a–c), there is an increase in the expression of 5-LOX enzyme after IR injury. To determine the cellular localization of 5-LOX expression, sections from ischemic brain after 24 h of reperfusion were subjected to immunohistochemistry with antibody against 5-LOX. 5-LOX expression co-localized with NSE, a neuron specific marker, implying that 5-LOX protein expression was increased in neurons after IR (Fig. 2c). The expression also co-localized in the microglia/macrophage, as seen by merging the 5-LOX and microglia/macrophage antigen RCA-1 (Fig. 2i). RCA-1 is not a widely used antigen for microglia. However, Rezaie et. al. have lately described this antigen as specific to microglia, detecting both developing and resting adult microglia [25]. A co-localization study of 5-LOX and GFAP, a marker for activated astrocytes, showed a few 5-LOX/GFAP positive cells (Fig. 2f). These observations indicate that 5-LOX protein is up regulated mainly in microglia/macrophage and neurons in the ipsilateral hemisphere after IR injury.

### Inhibition by BW-B 70C of iNOS protein expression and p65 translocation in brain after IR injury

**Figure 3:**
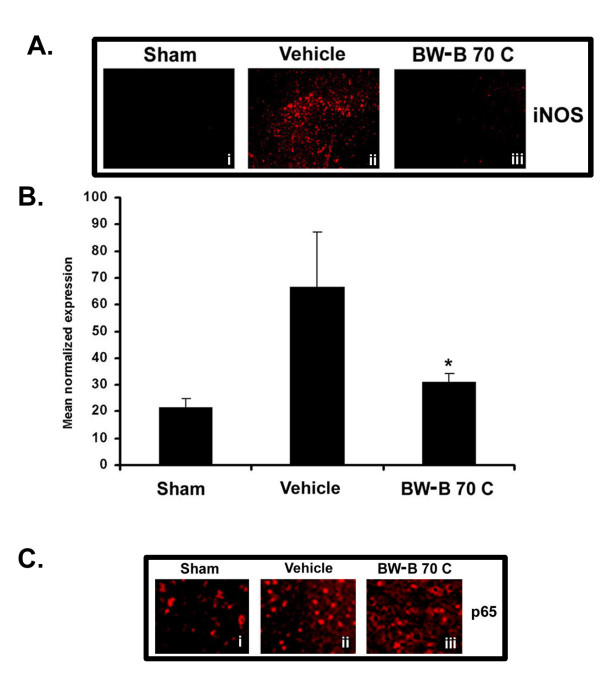
**BW-B 70C inhibits expression of iNOS and p65 *in vivo***. (A) Photomicrographs of immunohistochemistry of rat brain sections at 24 h reperfusion after 20 min MCAO showing remarkable iNOS expression in vehicle-treated (ii) but not in BW-B 70C-treated rats (iii). (B) BW-B 70C inhibited IR-induced iNOS gene expression measured as mRNA levels at 3 h of reperfusion after 20 min MCAO. The results are presented as mean ± SD of normalized expression of target gene with respect to GAPDH mRNA from three sets of animals. (C) Treatment with BW-B 70C prevented the nuclear translocation of p65 subunit of NF-κB (i-iii); the vehicle-treated animals showed nuclear translocation (ii) and treatment with BW-B 70C reversed this (iii) at 24 h reperfusion after 20 min MCAO. Figures are representative of similar results obtained from three groups of animals. A (i-iii) magnification 100 X; C (i-iii) magnification 200 X. *p < 0.01 vs. vehicle (n = 3).

As concluded from the data above (Figs. 1E and 2), there is an increased expression of 5-LOX enzyme in the brain after IR injury. Growing evidence suggests that 5-LOX and iNOS communicate and regulate the signaling cascade of inflammatory gene expression [11,26]. BW-B 70C treatment reduced the IR injury-induced inflammatory response by down-regulating iNOS expression (Fig. 3A iii) via inhibition of iNOS gene expression (Fig. 3B). This gene expression was quantified by real time PCR analysis of mRNA 3 h after reperfusion.

Activation of NF-κB is involved in iNOS gene expression and is associated with the translocation of p50:p65 heterodimer into the nucleus. BW-B 70C prevented nuclear translocation of p65 subunit *in vivo*, as demonstrated by immunohistochemistry (Fig. 3C, iii). Taken together, these data suggest that BW-B 70C leads to down-regulation of iNOS gene expression by inhibiting p65 translocation to the nucleus.

### BW-B 70C attenuates LPS-mediated expression of iNOS and levels of NO in BV2 microglial cell line

It has been documented that iNOS-derived NO from microglia/macrophages contributes to the pathobiology of cerebral IR injury [27]. Earlier we showed that glial cells, specifically microglia, produce NO in response to induction of iNOS by LPS and cytokines [28]. NO produced by iNOS has been shown to contribute to neuronal death in neurogenerative diseases [29]. Furthermore, growing evidence suggests the role of 5-LOX in induction of inflammatory genes [30]. Therefore, we examined the role of 5-LOX in the regulation of iNOS expression in glial cells *in vitro*.

BW-B 70C was not toxic up to 75 μM concentration as assessed *in vitro *by MTT assay (data not shown). The other selective 5-LOX inhibitor, AA-861 was found to be toxic at 50–75 μM concentrations and caused cell death. MTT assays were not performed on caffeic acid-treatment experiments because, while it is a potent 5-LOX inhibitor, it is not selective. Hence, we used BW-B 70C in cell culture experiments.

**Figure 4:**
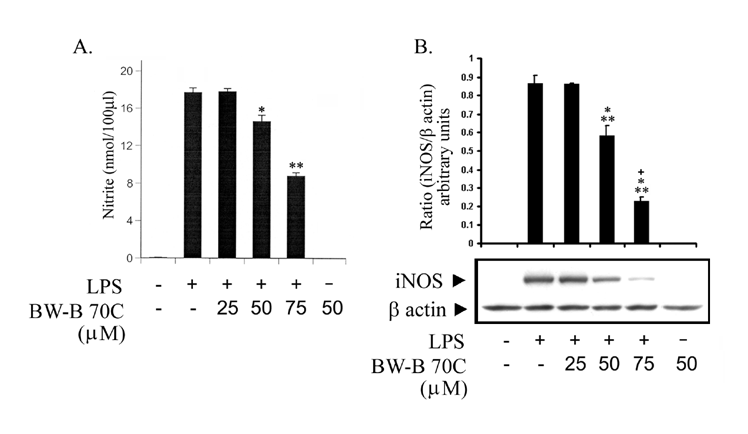
**BW-B 70C inhibits LPS-mediated iNOS expression in BV2 cells. **(A) BV2 Cells were pretreated for 30 min with different concentrations of BW-B 70C followed by LPS (1 μg/ml) treatment. After 24 h, NO as nitrite was quantified in supernatant by Griess reagent. Data are presented as means ± SD for 3 different experiments. (B) Cell lysates were processed for western blot analysis of iNOS and β-actin after 24 h of stimulation with LPS (1 μg/ml). Figures are representative of 3 different experiments. *p < 0.001 vs. LPS; **p < 0.001 vs. LPS+25 μM BW-B70C; ***p < 0.001 vs. LPS+25 μM BW-B70C; ^+^p < 0.001vs LPS + 50 μM BW-B 70C.

Treatment with BW-B 70C (up to 75 μM) attenuated NO production measured as nitrite (Fig. 4A) and iNOS protein expression (Fig. 4B) quantified by immunoblot analysis in LPS-treated cells. These data suggest that 5-LOX inhibition lead to the down-regulation of iNOS expression.

### BW-B 70C attenuates LPS-mediated expression of NF-κB luciferase activity in BV2 microglial cells

**Figure 5:**
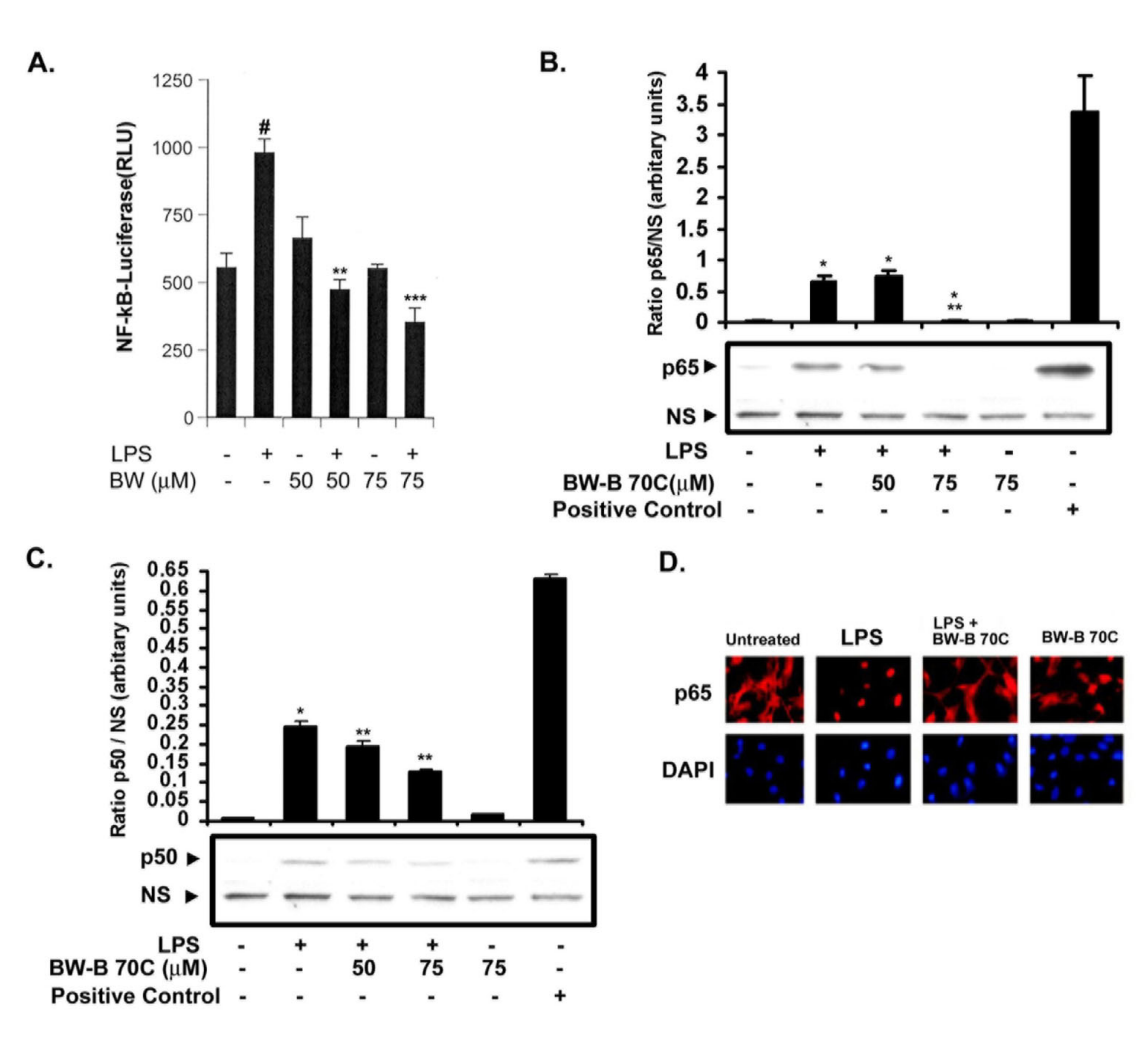
**BW-B 70C inhibits LPS-induced NF-κB activation in microglial BV2 cells and prevents nuclear translocation of p65 in rat primary microglia. **(A) Microglial cells (BV2) were transiently co-transfected with 1.5 μg of NF-κB luciferase reporter construct. Cells were pre-treated with BW-B 70C (25–75 μM) for 30 min followed by LPS (1 μg/ml) stimulation for 4 h. Luciferase activity was normalized with respect to β-galactosidase activity. Data are means ± SD of three different experiments. Immunoblot was performed at 1 h post treatment with LPS (1 μg/ml) for p65 (B) and p50 (C) in nuclear extract from primary microglia. A non-specific band (NS) was taken as internal standard. Blots are representative of three different experiments. (D) Immunohistochemcal analysis of rat primary microglia showing nuclear translocation of p65 1 h post LPS treatment. Cells were pretreated with BW-B 70C (75 μM) for 30 min before stimulation with LPS (1 μg/ml). Red fluorescence shows positive reaction for p65 and blue fluorescence showed nuclear staining with DAPI. LPS treatment translocated p65 to the nucleus, and treatment with BW-B 70C reversed it. Figures are representative of 3 experiments. (Magnification 200 X). ^#^p < 0.001 vs. untreated; *p < 0.001 vs. untreated; **p < 0.001 vs. LPS; ***p < 0.001 vs. LPS+BW-B 70C (50 μM).

Because the activation of NF-κB is important for the induction of iNOS in glial cells [31], the effect of BW-B 70C on the activation of NF-κB was examined in BV2 cells. BW-B 70C inhibited the LPS-mediated NF-κB-dependent luciferase activity in a dose-dependent manner (Fig. 5A).

To confirm further the inhibition of NF-κB by BW-B 70C, we examined the effect of BW-B 70C on nuclear translocation of p50/p65 complex in LPS-treated primary microglial cells. LPS stimulated the nuclear translocation of p65 and p50 subunits to the nucleus, and the translocation was prevented by BW-B 70C treatment (Fig. 5B–C). The observation was confirmed by immunocytochemical analysis (Fig. 5D). These results indicate that inhibition of 5-LOX prevents nuclear translocation of p50/p65 complex, which may cause down-regulation of NF-κB activity.

## Discussion

Brain damage caused by IR is due, in part, to secondary injury from inflammation [32]. The degree of inflammation is exacerbated by increased lipid peroxidation which increases neuronal death [33]. Leukotrienes derived from the metabolism of arachidonic acid by the 5-LOX enzyme are potent inflammatory mediators in IR injury [34]. Recently, 5-LOX has been shown to be involved in ischemic-like injury in neuronal PC12 cells [35]. As in human brain following stroke [7], we found increased expression of 5-LOX after IR in rat brain neurons and microglia/macrophage (Fig. 2).

Many substituted N-hydroxyureas, including BW-B 70C and Zileuton, are well-known potent and selective inhibitors of 5-LOX [36,37]. BW-B 70C has a long half-life and high oral bioavailability. Zileuton, an anti-leukotriene drug, has been identified as an anti-inflammatory compound [38] and is currently undergoing clinical trials in patients suffering from diseases in which leukotrienes play a pathogenic role [39]. To further support the idea that 5-LOX inhibition provides significant neuroprotection in experimental stroke, we used other well-established 5-LOX inhibitors, (caffeic acid and AA-861). The three 5-LOX inhibitors used in this study are structurally different. All were highly protective, reduced infarction and improved neurological deficit score (Fig. 1A–C and Table 2). 5-LOX inhibitors are therefore neuroprotective irrespective of their structural identities. BW-B 70C may be safer as a therapeutic agent due to the toxicity (cell viability assay using MTT) associated with AA-861 *in vitro *(data not shown). Furthermore, BW-B 70C may show greater potential in humans due to its structural similarity to Zileuton, high oral bioavailability and longer half life.

In our studies, we have used a focal cerebral ischemia animal model involving transient MCAO followed by reperfusion [28]. This model closely reproduces clinical ischemic brain damage, showing the oxidative stress and the inflammation observed in human ischemic stroke patients [40]. To test whether secondary inflammatory injury can be reversed by 5-LOX inhibitors, we selected a model with short-term (20 min) MCAO (with ~80% drop in CBF compared to basal value Fig. 1D) followed by reperfusion. In this model, there was more significant inflammatory response than cell death, which is appropriate to determine the efficacy of anti-inflammatory drugs [28].

NF-κB is one of the key regulators of inflammation. In brain ischemia, its roles in both cell survival and cell death have been shown [41]. Recently, Zhang et al [42] documented that activation of NF-κB in neurons is deleterious in cerebral ischemia. In addition, the NF-κB pathway has been shown to be instrumental in up-regulation of iNOS and of other inflammatory mediators that are damaging in stroke [28]. Inhibition of iNOS expression in microglia/macrophage by antioxidants has been shown to protect the brain after IR injury [43]. We demonstrated in this study that IR injury caused an increased expression of 5-LOX, NF-κB and iNOS. The attenuation of expression of these inflammatory mediators by 5-LOX inhibitors resulted in neuroprotection as seen by decreased infarction size and improved neurological deficit scores (Fig. 1A–C and Table 2). These inhibitors did not significantly alter physiologic parameters as shown in Table 1.

The critical aspect of 5-LOX/NF-κB signaling may be its direct interaction with the p65 subunit of NF-κB [14]. It has been reported that NF-κB activation occurs via 5-LOX-mediated generation of ROS in lymphoid cells [9]. However, the mechanisms of NF-κB activation by direct interaction with 5-LOX remain to be investigated. The 5-LOX inhibitor BW-B 70C used in this study inhibited NF-κB luciferase activity *in vitro *(Fig. 5A). It also inhibited p65 translocation to nucleus both *in vitro *(Fig 5B and 5D) and *in vivo *(Fig. 3C, iii). Furthermore, BW-B 70C reduced iNOS expression *in vitro *(Fig. 4B). We hypothesize that 5-LOX action is upstream to NF-κB, and its inhibition results in reduced expression of NF-κB and iNOS, thereby ameliorating inflammation.

The 5-LOX-leukotriene pathway has been implicated in aneurysm formation [44]. The present study accords with other studies showing neuroprotective effects against inflammation-mediated injury [45]. Inhibition of phospholipase A_2 _(PLA_2_), the enzyme that liberates arachidonic acid from membrane phospholipids, provides limited protection in stroke because the metabolism of polyunsaturated fatty acids (PUFA) is required to generate several anti-inflammatory eicosanoids [46] and epoxides [47]. The COX-2 pathway has been documented as a valuable therapeutic target for ischemic brain injury [48,49]. However, it is also recognized that chronic inhibition of this pathway produces an eicosanoid synthesis imbalance that promotes undesirable vascular effects [50]. Furthermore, the inhibition of the COX-2 pathway would result in the bioavailability of PLA_2_-derived PUFAs to lipoxygenases. This may result in excessive production of leukotrienes and the generation of ROS. Increased leukotriene production results in depletion of GSH, thus making cells vulnerable to ROS injury [51]. Inhibiting the 5-LOX/NF-κB pathway has potential to circumvent the problems associated with blocking PLA_2 _or the COX-2 pathway. However, it has been demonstrated that 5-LOX knock out mice do not show protection during IR [52]. In this context, we hypothesize that 5-LOX expression modulates multiple events, and its role may become evident only under pathological conditions. Further, a certain basal activity of the enzyme may be required physiologically, and the complete depletion of the enzyme is detrimental, as is the case with TNF-α knock out mice [53].

## Conclusion

This study demonstrates the protective effect of 5-LOX inhibitors when administered either prior to ischemic insult or at reperfusion in a rat model of experimental stroke. The protective effect of 5-LOX inhibitors is due in part to down-regulation of iNOS via inhibition of NF-κB activation. Acute stroke is a multi-component inflammatory disorder, and its treatment will involve agents antagonizing multiple mechanisms of inflammation. Therefore, we propose that 5-LOX inhibitors may have therapeutic potential to treat neuroinflammation in ischemic stroke injury.

## Competing interests

The author(s) declare that they have no competing interests.

## Authors' contributions

This study is based on an original idea of Mushfiquddin Khan (MK) and Inderjit Singh. MK and Manu Jatana (MJ) wrote the manuscript. Shailendra Giri directed and performed the *in vitro *experiments. Elango Chinnasamy, Manu Jatana and Mubeen A Ansari carried out the animal studies. Avtar K Singh critically examined the immunohistochemical studies.
